# PacBio sequencing of human fecal samples uncovers the DNA methylation landscape of 22 673 gut phages

**DOI:** 10.1093/nar/gkad977

**Published:** 2023-10-30

**Authors:** Yanqiang Ding, Liuyang Zhao, Guoping Wang, Yu Shi, Gang Guo, Changan Liu, Zigui Chen, Olabisi Oluwabukola Coker, Junjun She, Jun Yu

**Affiliations:** Institute of Digestive Disease and Department of Medicine and Therapeutics, State Key Laboratory of Digestive Disease, Li Ka Shing Institute of Health Sciences, CUHK-Shenzhen Research Institute, The Chinese University of Hong Kong, Hong Kong SAR, China; Institute of Digestive Disease and Department of Medicine and Therapeutics, State Key Laboratory of Digestive Disease, Li Ka Shing Institute of Health Sciences, CUHK-Shenzhen Research Institute, The Chinese University of Hong Kong, Hong Kong SAR, China; Institute of Digestive Disease and Department of Medicine and Therapeutics, State Key Laboratory of Digestive Disease, Li Ka Shing Institute of Health Sciences, CUHK-Shenzhen Research Institute, The Chinese University of Hong Kong, Hong Kong SAR, China; Institute of Digestive Disease and Department of Medicine and Therapeutics, State Key Laboratory of Digestive Disease, Li Ka Shing Institute of Health Sciences, CUHK-Shenzhen Research Institute, The Chinese University of Hong Kong, Hong Kong SAR, China; Center for Gut Microbiome Research, Department of Surgery, Med-X Institute, Department of High Talent, The First Affiliated Hospital of Xi’an Jiaotong University, Xi’an, China; Institute of Digestive Disease and Department of Medicine and Therapeutics, State Key Laboratory of Digestive Disease, Li Ka Shing Institute of Health Sciences, CUHK-Shenzhen Research Institute, The Chinese University of Hong Kong, Hong Kong SAR, China; Department of Microbiology, The Chinese University of Hong Kong, Hong Kong SAR, China; Institute of Digestive Disease and Department of Medicine and Therapeutics, State Key Laboratory of Digestive Disease, Li Ka Shing Institute of Health Sciences, CUHK-Shenzhen Research Institute, The Chinese University of Hong Kong, Hong Kong SAR, China; Center for Gut Microbiome Research, Department of Surgery, Med-X Institute, Department of High Talent, The First Affiliated Hospital of Xi’an Jiaotong University, Xi’an, China; Institute of Digestive Disease and Department of Medicine and Therapeutics, State Key Laboratory of Digestive Disease, Li Ka Shing Institute of Health Sciences, CUHK-Shenzhen Research Institute, The Chinese University of Hong Kong, Hong Kong SAR, China

## Abstract

Gut phages have an important impact on human health. Methylation plays key roles in DNA recognition, gene expression regulation and replication for phages. However, the DNA methylation landscape of gut phages is largely unknown. Here, with PacBio sequencing (2120×, 4785 Gb), we detected gut phage methylation landscape based on 22 673 gut phage genomes, and presented diverse methylation motifs and methylation differences in genomic elements. Moreover, the methylation rate of phages was associated with taxonomy and host, and N6-methyladenine methylation rate was higher in temperate phages than in virulent phages, suggesting an important role for methylation in phage-host interaction. In particular, 3543 (15.63%) phage genomes contained restriction-modification system, which could aid in evading clearance by the host. This study revealed the DNA methylation landscape of gut phage and its potential roles, which will advance the understanding of gut phage survival and human health.

## Graphical Abstract

**Graphical Abstract:**
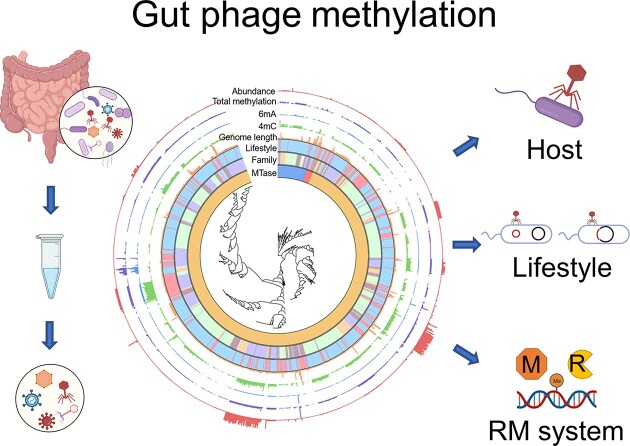

## Introduction

Gut phages have an important impact on gut ecology and human health (1,2). However, previous studies on gut phages mainly focused on abundance, with few information on phage methylation modifications. In particular, the methylation landscape of gut phages is largely unknown.

Methylation plays key roles in phage-host interaction, gene expression regulation, replication and virulence for phages. Unlike the main methylation type (5-methylcytosine (5mC)) in eukaryotes, N6-methyladenine (6mA) and N4-methylcytosine (4mC) are the two predominant methylation types in prokaryotes and phages (3–5). As a means of defence, the host can recognise unmethylated motifs of foreign phage DNA through the restriction-modification (RM) systems (6) for degradation and clearance. On the other hand, phage can evade host's clearance by methylating special recognition motifs (7). DNA methylation in phages has also been implicated in the regulation of life cycle. For instance, the G***A***TC motif methylation of *rha* antirepressor gene in phage *Vibrio harveyi myovirus like* (VHML) is required for switching between lysogenic cycle, where the phage lay dormant as part of the genome of infected cells, and lytic cycle, with rapid replication and eventual lysis of host cells (8,9).

Further investigation of methylation in phages is therefore warranted. This is especially important in the gut, where the association of gut phages with human health is becoming increasingly evident (1). With extensive antibiotics use leading to the emergence of drug-resistant bacteria, using phage therapy to specifically target and lyse pathogenic bacteria to combat infections has received rising attention (10). In this respect, phage DNA methylation represents an adaptive response to bacteria host RM system, contributing to phage fitness. A deeper understanding of gut phage methylation and its roles can, in the future, lead to greater control of gut phages and their safe use as probiotics and phage therapy in human health.

Phage methylation studies are sparse mainly due to technical limitations. The early virus methylation studies mainly focused on cultivated and purified RNA viruses with complex and low-throughput methods (11,12). With the introduction of third-generation sequencing technologies, such as PacBio single-molecule real-time (SMRT) and Oxford Nanopore, the possibility to detect DNA methylation patterns of uncultivated virus in a high-throughput manner (3,4,13,14) has been enabled. In particular, 6mA and 4mC can be reliably detected through PacBio sequencing (3,4).

To reveal the methylation landscape of gut phages, we leveraged a workflow based on virus-like particles (VLPs) enrichment and PacBio sequencing, and analysed the relationships between phage methylation and their taxonomy, host, lifestyle, and RM system. These findings are instructive for future gut phage epigenetics and human health research.

## Materials and methods

### Sample collection and virus-like particles DNA sequencing

**Figure 1.**
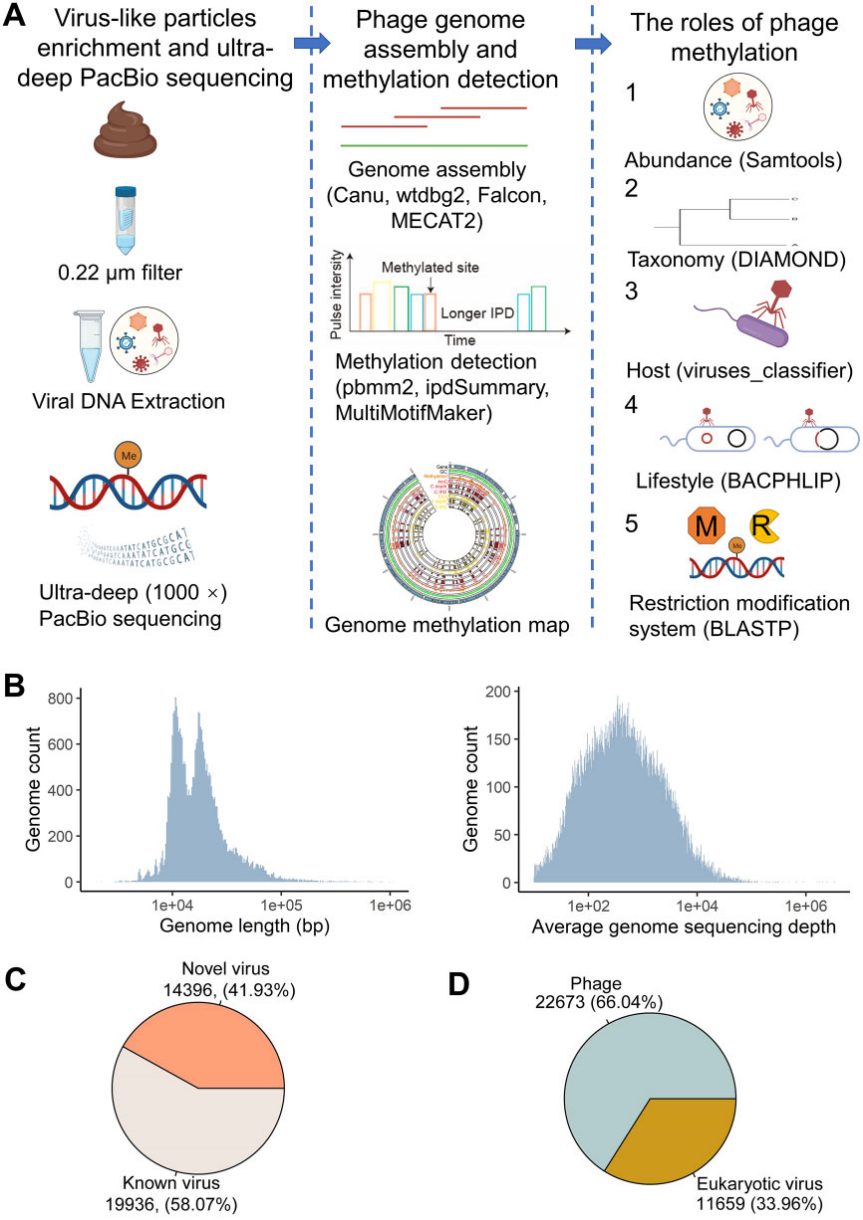
Workflow for detecting gut phage methylation signals based on virus-like particles (VLPs) enrichment and PacBio sequencing. (**A**) The workflow of this study mainly consisted of three parts: VLPs enrichment and PacBio sequencing, phage genome assembly and methylation detection, and the roles of phage methylation; (**B**) virus genome assembly results with the average assembled genome length of 22948 bp and the average genome sequencing depth of 2120×; (**C**) the proportion of novel virus genomes in all assembled virus genomes; (**D**) the proportion of phage genomes in all assembled virus genomes.

This study was approved by Joint Chinese University of Hong Kong-New Territories East Cluster Clinical Research Ethics Committee. Subjects signed informed consent before sample collection. Stool samples were collected from seven healthy individuals (age: 52.7 ± 11.5) in Hong Kong. VLPs (>10^10^/sample) were enriched from the freshly collected stool samples by filtering with 0.45 and 0.22 μm polyvinylidene fluoride membrane filters (Millipore, Burlington, MA), followed by filtering with Amincon Ultra 50 ml–50 kDa molecular weight cutoff filter (Millipore) as previously described (15). Circular consensus sequencing was performed on the viral DNA (length > 15 kb, 10 μg/sample) on PacBio Sequel II System (15 kb-fragment sequencing libraries, 1 sample per PacBio cell, Single-Molecule Real-Time Cell 8M Tray, Pacific Biosciences, Menlo Park, CA) without PCR amplification (Figure 1A).

### Virus genome assembly

Raw PacBio sequencing reads were filtered using pbccs (version 4.1) with ‘min-passes 3, min-rq 0.90’ parameter. The sequencing reads were aligned to the human genome GRh38 using minimap2 (version 2.17-r941) to remove human reads (16). Filtered PacBio long reads were subjected to virus genome *de novo* assembly with four methods: Canu (read correction, read trimming and contig construction) (17), wtdbg2 (18), Falcon (19) and MECAT2 (20). The assembled contigs were polished using Racon. The virus genomes were further identified from complex contigs using three methods: (i) Virsorter2 following the recommended quality control with default score cutoff and quality check step using CheckV (21,22); (ii) Deepvirfinder with *P* value <0.05 and length of genome >3000 bp (23) and (iii) VIBRANT with default parameters (except for length of genome > 3000 bp) (24). Only contigs that met at least two criteria were used for subsequent analysis. The assembled contigs were combined together, and redundancy was removed using the rapid genome clustering method based on pairwise average nucleotide identity (0.98) (22). The assembled contigs were further filtered as follows: (i) contigs with less than three virus genes per 10 kb were filtered out through predicting genes using Prodigal (version 2.6.3) and aligning to the Uniprot database (released March 2022) using Blast (version 2.2.31) with default parameters; (ii) contigs with bacterial sequences were filtered out through aligning to the NCBI database (released April 2022) using Blast (version 2.2.31) with default parameters; (iii) contigs with bacterial ribosomal RNA were filtered out using barrnap (version 0.9); (iv) contigs with plasmid were filtered out through aligning to the PLSDB (released February 2022) and NCBI plasmid database (released March 2022) using Blast (version 2.2.31) with default parameters; (v) contigs with <90% coverage were filtered out through aligning the reads to contigs using minimap2 (version 2.17-r941). The virus genome assembly quality was further checked by CheckV (22), Deepvirfinder (23) and VIBRANT (24). The results of these three methods were combined in the final evaluation of virus genomes.

The virus genomes were then aligned to the combined NCBI (released April 2022), the Metagenomic Gut Virus (MGV) catalog and the human Gut Virome Database (GVD) reference virus databases using Blast (version 2.2.31) with default parameters. The average nucleotide identity between the assembled virus genome and the reference virus genome was calculated with fastANI. Novel virus genomes were identified with threshold of 95% average nucleotide identity and over 85% alignment fraction (25).

The relative abundances of viruses were estimated by aligning the sequencing reads to the assembled virus genomes using pbmm2. The depth for each virus was calculated by Samtools (version 1.10) (26) and bamcov, and was normalized by virus genome length. The relative abundance of each virus was calculated as the depth of the virus normalized by the total number of reads for each sample (27).

### Methylation signal detection

High-quality PacBio long reads were aligned to the assembled phage genomes using pbmm2 with the SUBREAD alignment modes. The methylation states of nucleic acids were detected using ipdSummary.py. The reads coverage required to call a modified base was at least 10×. The methylation fraction was estimated along with 95% confidence interval bounds when the reads coverage ≥10×. Modification types, 4mC and 6mA were identified with reads coverage ≥5×. 5mC was not explored because it is rare in prokaryotes and phages (3–5). The *P* value cutoff was 0.01. Nucleic acids methylation motifs were identified by MultiMotifMaker with score ≥30 (28). The methylation states of nucleic acids were reprocessed after methylation motifs were identified. Putative methyltransferase recognition motifs were further identified by MEME (Version 5.4.1) (29). R packages ggseqlogo and circlize were used respectively to plot the logos of putative methyltransferase recognition motifs and the phage genome methylation maps.

To evaluate the effect of sequencing depth on methylation signal detection, the PacBio sequencing data was randomly sampled to 1000, 750, 500, 250, 125, 50 and 10 Gb. The sequencing depth for each phage was calculated using pbmm2 and bamcov with different amounts of sequencing data. Methylation states of nucleic acids were then detected following the pipeline described above.

### Virus taxonomic annotation

Virus taxonomy was annotated using PhaCGN2.0, a GCN based model for virus taxonomy classification, with default parameters (30). The significant differences in methylation rates (total methylation rate, 4mC and 6mA) among different clusters were evaluated using Wilcoxon rank-sum test in R 4.0.3.

### Virus host prediction

Whether a virus infects eukaryotes or bacteria/archaea was determined using the quadratic discriminant analysis by viruses_classifier (31). Human-infecting viruses were further identified using the k‐nearest neighbor model trained on the virus genomes by humanVirusFinder (32). Bacteria/archaea hosts of identified phages were predicted by iPHoP (33). Phage-host interaction with confidence score >90 was considered a reliable prediction. Cumulative distribution analysis was performed to compare differences in the total methylation rate between phages from the top 2 hosts in each sample. Significant differences in total methylation rates were evaluated using the Kolmogorov–Smirnov test in R 4.0.3.

### Phage lifestyle prediction

The phage lifestyles (i.e. virulent or temperate) were predicted from conserved protein domains via a Random Forest classifier, BACPHLIP with a score cutoff of 0.8 (34,35). Differences in methylation rates (total methylation rate, 4mC and 6mA) between different lifestyles were evaluated using Wilcoxon rank-sum test in R 4.0.3.

### Phage restriction-modification (RM) system

Phage RM system was predicted through homology comparison with reference database (36). The reference database was built based on the experimentally characterized ‘Gold Standard’ methyltransferases, endonucleases and specificity subunit sequences downloaded from New England Biolabs’ REBASE database (37). For each phage, the protein sequences were aligned to the reference database using BLASTP with default parameters, implementing BLOSUM62 similarity Matrix, a Compositionally Adjusted Substitution Matrices method. An enzyme homolog was identified using two strict thresholds: the total alignment length >75% of the query length and *e* value less than 1e–15. BLASTP results with the highest scores were used for further analysis. Phage RM system heatmap analysis was performed using ComplexHeatmap package in R 4.0.3. Significant differences in methylation rates (total methylation rate, 4mC and 6mA) between the phages with and without RM system were evaluated using Wilcoxon rank-sum test in R 4.0.3.

### Statistical analysis

Significant differences were evaluated using Wilcoxon rank-sum test, Fisher's exact test or Kolmogorov–Smirnov test in R 4.0.3. Correlations between phage methylation rate (total methylation rate, 4mC methylation rate and 6mA methylation rate) and relative abundance were calculated with Spearman method, and the smoother line was added using linear smooths method in R 4.0.3. *P* < 0.05 was considered statistically significant.

## Results

### A total of 22 673 gut phage genomes were obtained

After a series of refined VLPs enrichment processes as previously described (15), extracted high-quality VLP DNA from the faecal samples of seven healthy adults was sequenced using PacBio Sequel II System (Figure 1A). A total of 4785 Gb of long reads, with an average of 684 Gb per sample, was generated. After filtering by pbccs, 14 281 162 high quality long reads (Q30 > 99.99%) were obtained, with an average of 2 040 166 reads per sample. The average read length ranged from 7068 to 13 998 bp in the seven samples (Supplementary Figure S1A and B, Supplementary Table S1).

After assembling and filtering, a total of 34 332 virus genomes were obtained. The average assembled genome length was 22 948 bp with an average genome sequencing depth of 2120× (Figure 1B). The average gene number was 30 per virus genome (Supplementary Figure S1C). Moreover, as compared with known virus genome databases (NCBI (released April 2022), MGV and GVD), 14396 novel virus genomes were found, accounting for 41.93% of the total virus genomes assembled in this study (Figure 1C). We further performed virus host characterization analysis and obtained 22 673 phages, accounting for 66.04% of all viruses (Figure 1D). The phage genomes were used for downstream methylation analysis.

### PacBio sequencing detected methylation signals

**Figure 2.**
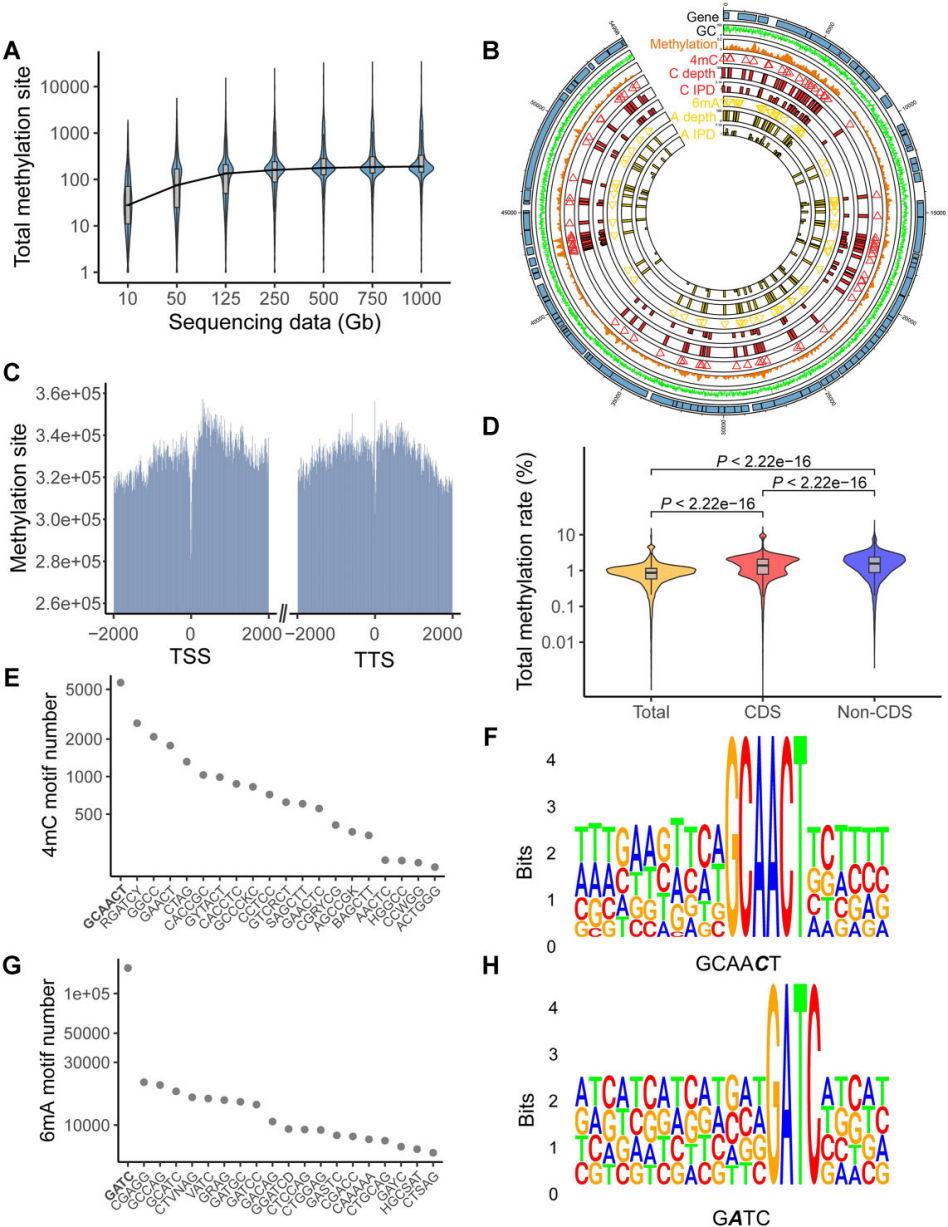
Genome methylation map and methylation motifs of gut phages. (**A**) the total methylation site number detected with different amounts of sequencing data; (**B**) the genome methylation map of phage H1_phage1, from inside to outside: 6mA inter-pulse duration (IPD), 6mA coverage depth, 6mA methylation site, 4mC IPD, 4mC coverage depth, 4mC methylation site, total methylation site, GC content and coding gene; (**C**) the methylation site number around the transcription start site (TSS) and transcription termination site (TTS) was lower than that of gene body and other regions in the phage genomes; (**D**) the total methylation rate was significantly higher in non-CDS regions than in CDS regions; (**E**) the number of top 20 4mC methylation motifs in gut phages; (**F**) the most common 4mC methylation motif (GCAA***C***T) in gut phages; (**G**) the number of top 20 6mA methylation motifs in gut phages; (**H**) the most common 6mA methylation motif (G***A***TC) in gut phages. Italic bold represents the methylated base.

We assessed methylation signal detection under different sequencing depths. We observed that the coverage depth of the phage genomes increased as the amount of sequencing data increased (Supplementary Figure S2A). The minimum sequencing depth for methylation signal detection recommended by PacBio is 25×. Only 16.24% of the phage genomes had a sequencing depth higher than 25× with 10 Gb sequencing data, while 82.1% did with 1000 Gb sequencing data. (Supplementary Figure S2A). As the sequencing data and sequencing depth increased, the number of detected methylation sites also gradually increased and reached a plateau between 500 and 1000 Gb (Figure 2A, Supplementary Figure S2B and C). Together, these results reveal that increased sequencing depth promoted the detection of methylation signals, and that ultra-deep PacBio sequencing could detect as many methylation signals as possible with current technology in this study.

### Genome methylation map and methylation motifs of gut phages

To determine the methylation patterns in gut phages, we detected methylation sites and types based on the inter-pulse duration (IPD). Methylation signals were detected in 21717 phages, accounting for 95.78% of all phages. For each phage, we obtained a genome methylation map with 6mA IPD, 6mA coverage depth, 6mA methylation sites, 4mC IPD, 4mC coverage depth, 4mC methylation sites, total methylation sites, GC content and gene coding information (Figure 2B; Supplementary Figure S3). We found that the methylation around the predicted transcription start site and transcription termination site was lower significantly than that of gene body and other regions in the phage genomes (Figure 2C, *P* < 2.2e–16). The total methylation rate, 4mC methylation rate and 6mA methylation rate were significantly higher in non- coding DNA sequences (CDS) regions than in CDS regions (*P* < 2.2e–16) (Figure 2D; Supplementary Figure S4).

The total methylation rate of the phages varied greatly from 0 to 14.90% with an average rate of 0.92%. The 4mC methylation rates ranged from 0 to 13.55%, and the 6mA methylation rates ranged from 0 to 41.98%. The putative methyltransferase recognition motifs were identified by MEME (Version 5.4.1). Motif GCAA***C***T (*e* value < 1e–10, *n* = 5662) was the most identified 4mC motif overall (Figure 2E and F), followed by other identified 4mC motifs, such as RGAT***C***Y (*n* = 2676), GG***C***C (*n* = 2084), GAA***C***T (*n* = 1773), ***C***TAG (*n* = 1316) (Figure 2E). Motif G***A***TC (*e* value < 1e–10, *n* = 156 423) was the most identified 6mA motif overall (Figure 2G and H), followed by other identified 6mA motifs, such as CG***A***GG (*n* = 21 192), GCC***A***G (*n* = 20 170), GC***A***TC (*n* = 18 073) (Figure 2G).

### The methylation rates of gut phages varied with taxonomy

**Figure 3.**
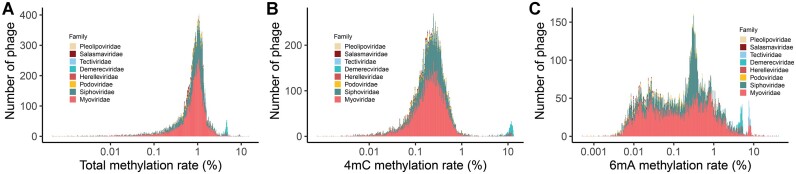
Phage methylation varied widely among families. The total methylation rate (**A**), 4mC methylation rate (**B**) and 6mA methylation rate (**C**) distribution of phages in family level, and the methylation rates varied among families.

We further explored the relationship between phage methylation and taxonomy. We found that most of the gut phages were within the class *Caudoviricetes*. The methylation rates of gut phages varied with the taxonomy. The total methylation rates of *Myoviridae* phages (median 0.87%) were significantly different from those of *Siphoviridae* phages (0.90%, *P* = 0.02) (Figure 3A). The 4mC methylation rates of *Myoviridae* phages (0.26%) were significantly different from those of *Siphoviridae* phages (0.34%, *P* = 0.003) (Figure 3B), and the 6mA methylation rates of *Myoviridae* phages (0.10%) were also significantly different from those of *Siphoviridae* phages (0.24%, *P* = 4.6e–8) (Figure 3C). These results indicate that gut phage methylation is related to their taxonomy.

### The methylation rates of phages were associated with host

The hosts of the phages were further determined. The number of phages with predicted host was 12 665, while the number of phages without predicted host was 10 008. At the family level, Bacteroidaceae was the most common bacterial host, while *Bacteroides* and *Faecalibacterium* were the most common bacterial hosts at the genus level. The predicted interactions between the phages and their hosts varied widely among samples (Supplementary Figure S5). Moreover, phages from various hosts had significantly different overall methylation rates (*P* < 0.05) (Supplementary Figure S6). These results indicate that phage methylation is associated with the host.

### Temperate phages had higher 6mA methylation rate than virulent phages

**Figure 4.**
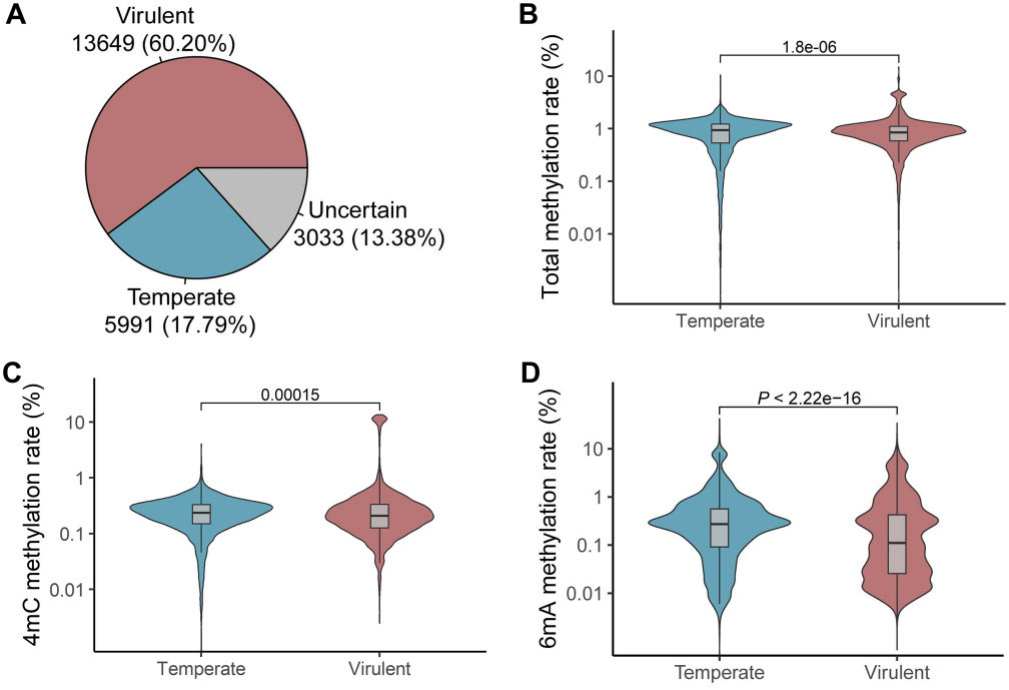
Phage methylation is associated with its lifestyle. (**A**) The proportion of temperate and virulent phages in all phages; (**B**) lower total methylation rate in temperate phages than in virulent phages; (**C**) the 4mC methylation rate in temperate and virulent phages; (**D**) higher 6mA methylation rate in temperate phages than in virulent phages.

To determine the role of methylation in phage lifestyle, we used BACPHLIP to predict the lifestyles of phages, and compared the methylation rates of phages with the lifestyles. A total of 5991 (17.79%) phages were temperate, and 13 649 (60.20%) phages were virulent (Figure 4A). The total methylation rate of temperate phages (0.86%) was significantly lower than that of virulent phages (0.94%) (*P* = 1.8e–06) (Figure 4B). The average 4mC methylation rate of temperate phages was 0.23%, which was significantly lower than that of virulent phages (0.55% in average) (*P* = 0.00015) (Figure 4C). However, the 6mA methylation rate of temperate phages (0.58% in average) was significantly higher than that of virulent phages (0.47% in average) (*P* < 2.2e–16) (Figure 4D). Moreover, we found that the main 6mA methylation motif of *rha* antirepressor gene was G***A***TC (Supplementary Figure S7).

### Methylation rates were higher in phages with restriction-modification (RM) system than in phages without RM

**Figure 5.**
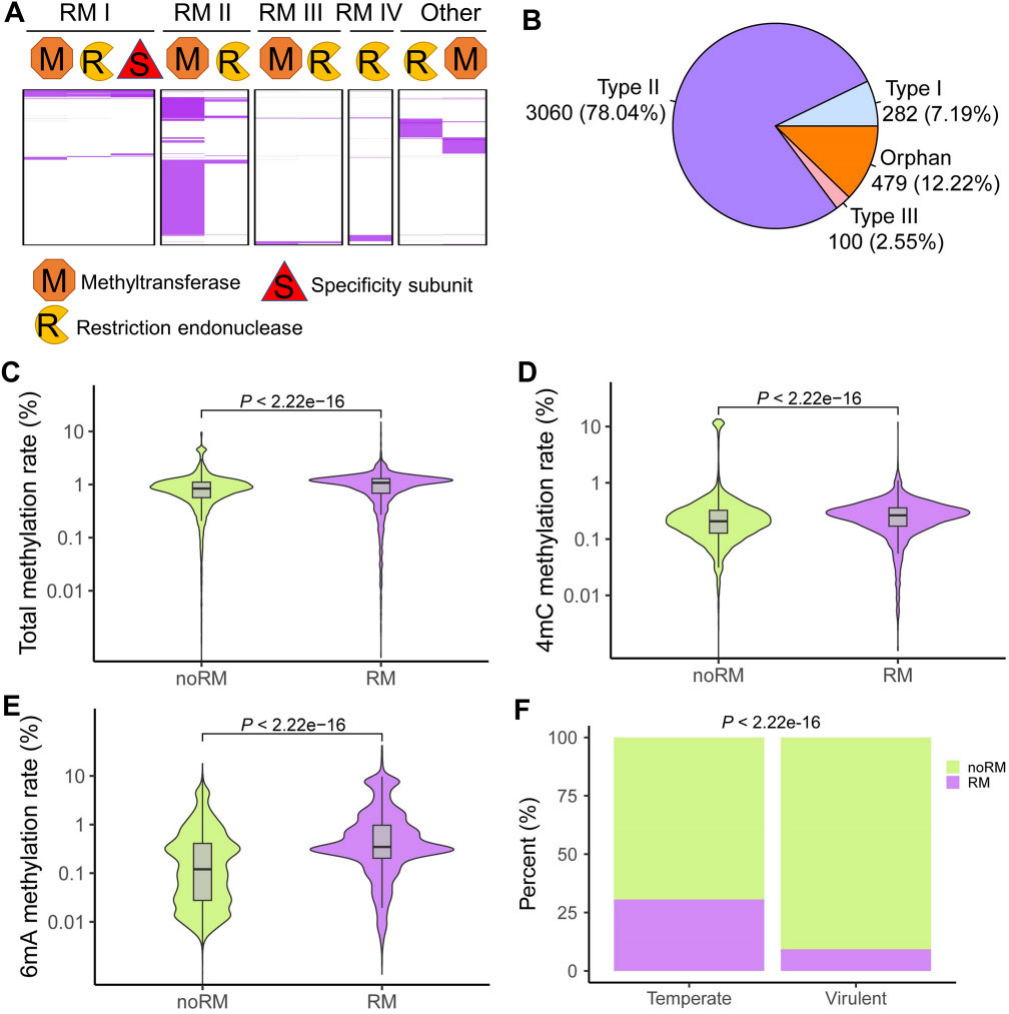
Phage restriction-modification (RM) system and methylation. (**A**) The predicted methyltransferases, restriction endonucleases and specificity subunits of RM system in gut phages, including RM type I, RM type II, RM type III and RM type IV; (**B**) the proportion of predicted type I, type II, type III and orphan methyltransferases; (**C–E**) higher total methylation rate, 4mC methylation rate and 6mA methylation rate in gut phages with RM than in gut phages without RM; (**F**) the proportion of the RM system in temperate phages was significantly higher than that in virulent phages.

We further investigated the role of methylation in phage RM system. The phage RM system was predicted based on the ‘Gold Standard’ from New England Biolabs’ REBASE database. Subunits related to the RM system were found in 3543 phage genomes, accounting for 15.63% of all phages (Figure 5A). A total of 3921 methyltransferases were found in the phage genomes. The most prevalent methyltransferase type was type II methyltransferase (*n* = 3060), accounting for 78.04%. This was followed by orphan methyltransferase (*n* = 479), accounting for 12.22%. Type I (*n* = 282) and type III (*n* = 100) methyltransferases accounted for 7.19% and 2.55%, respectively (Figure 5B). We found that many of the identified RM systems were incomplete. For example, most of the type II modification systems only had methyltransferases, but lacked restriction endonucleases (Figure 5A).

The total methylation rate of phages with RM was 0.97% in average, which was significantly higher than that of phages without RM (0.91% in average) (*P* < 2.2e–16) (Figure 5C). The 4mC methylation rate of phages with RM was also significantly higher than that of phages without RM (*P* < 2.2e–16) (Figure 5D). The average 6mA methylation rate of phages with RM was 1.16%, which was significantly higher than that of phages without RM (0.39% in average) (*P* < 2.2e–16) (Figure 5E). In addition, we found that the proportion of RM system in temperate phages (30.56%) was significantly higher than that in virulent phages (9.20%) (*P* < 2.2e–16) (Figure 5F).

## Discussion

Phages employ DNA methylation as an epigenetic modification mechanism to control replication, gene expressions, and interaction with the host. Taking advantage of enriched VLPs and PacBio sequencing, we detected methylation sites based on 22 673 assembled gut phage genomes in this study. We found that methylation rates vary with the taxonomy, host, lifestyle and RM system of gut phages.

Ultra-deep sequencing based on a large number of gut phage genomes could reveal the methylation landscape of gut phages. A previous study had used Oxford Nanopore technology to detect 5mC and 6mA methylation in five gut viruses with 10× depth (14). A recent study investigated phage 4mC and 6mA methylation through PacBio sequencing with 412× depth, which may present only a part of the global picture of phage DNA methylation as discussed (38). In our study, ultra-deep (2120×) PacBio sequencing was used to detect 4mC and 6mA methylation, and the number of detected methylation sites reached a maximum with increased sequencing depth, showing that as many methylation signals as possible with current technology were detected under the ultra-deep PacBio sequencing.

We found that methylation around the transcription start sites and transcription termination sites were lower than that of the gene body and other regions in the phage genomes. The methylation rates were significantly higher in non-CDS regions than in CDS regions. This is similar to the low methylation of transcription start sites in eukaryotes (39). It is generally believed that DNA methylation before the transcription start site or around the transcription termination site tended to result in gene-silencing or read-through events (40,41). After phage DNA entering host cells, methylation might play an important role in phage gene expression regulation.

The main DNA methylation type in eukaryotes is 5mC, while the main methylation type in prokaryotes is 6mA (3). Our study identified 6mA and 4mC methylation types in gut phages. Not much research has been done on the function of phage 6mA methylation, and even less on the function of phage 4mC methylation. Previous studies on bacteria reported that 4mC methylation contributes to the genomic stability of *Deinococcus radiodurans* (42), and is critical for the virulence and epigenetic regulation of *Leptospira interrogans*, a human pathogen (43). Further research is needed to explore the implication of 4mC and 6mA methylation and motifs in gut phages.

The recognition motifs of phage methyltransferases are very diverse, and many of them were not found in the REBASE database (37). The most common 4mC motifs observed in this study was GCAA***C***T, followed by GG***C***C. For 6mA, the most common motif was G***A***TC overall. G***A***TC motif is the recognition motifs of methyltransferase Dam and restriction endonucleases Dpn I, Dpn II and Sau3A (44). Dam methylation controls the initiation of replication at G***A***TC containing promoters of bacteria such as *Vibro cholerae*, *Caulobacter cresentus* and reduces the frequency of transformation in other bacteria (44). It is possible that the conserved G***A***TC 6mA methylation motif in phages contributes to the control of phages replication and evasion of host's endonuclease clearance.

We further explored the relationship between phages lifestyle and methylation. We found that 6mA methylation rate of temperate phages was higher than that of virulent phages, whereas 4mC methylation rate was the opposite, suggesting that 6mA and 4mC may play different roles in phages of different lifestyles. This result was consistent with previous study (38). 6mA plays key roles in the regulation of RM system, DNA replication and gene expression, which can affect the survival of temperate phages in the host (45,46). Furthermore, we confirmed that the main 6mA methylation motif of *rha* antirepressor gene was G***A***TC, which was consistent with the study on phage VHML (8,9). These results suggest an important role of methylation in the lifestyles of phages.

Host restriction endonucleases can cleave foreign phage DNA, through the RM system, only if the recognized motif is unmethylated (6). Meanwhile, phages can modify the special motifs through methylation to evade clearance by the host RM system (7). In this study, subunits related to the RM system, including 3921 methyltransferases, were found in 3543 phage genomes. In particular, the 6mA methylation rate of phages with RM was 3 times as high as that of phages without RM. This was consistent with a previous report that 6mA was widespread in RM system (47). In addition, we found that the proportion of the RM system in temperate phages was significantly higher than in virulent phages. When temperate phages genome integrates into the host genome, the phage RM system can protect it from the host's RM system's restriction enzyme clearance (45). Our results suggest that phages may use their methyltransferase to modify the shared recognized motifs (for example, G***A***TC motif) with 6mA methylation to evade clearance by the host's RM system.

In conclusion, this study revealed the DNA methylation landscape of gut phages through VLPs enrichment and PacBio sequencing. Based on 22673 assembled gut phage genomes, we presented the methylation differences in phage genomic elements and diverse 4mC and 6mA methylation motifs. Furthermore, we found that phage methylation is associated with phage taxonomy, the host, lifestyle, and evading the host's RM system clearance. This study disclosed the DNA methylation landscape of gut phages, which will expand our understanding of the survival mechanisms of gut phages and their potential clinical application.

## Supplementary Material

gkad977_supplemental_files

## Data Availability

The PacBio sequencing reads are available in the NCBI at https://www.ncbi.nlm.nih.gov/bioproject under the accession code PRJNA905328. The detailed methylation information is available in Figshare at https://doi.org/10.6084/m9.figshare.22580554.
