# Supplementary material for: PacBio sequencing of human fecal samples uncovers the DNA methylation landscape of 22 673 gut phages
Source: Nucleic Acids Res. 2023 Oct 30;51(22):12140–9. doi: 10.1093/nar/gkad977 (PMC10711547; doi:10.1093/nar/gkad977)
Supplement: gkad977_supplemental_files [file gkad977_supplemental_files.zip › S Table 1.docx]

**Table S1. Ultra-deep PacBio sequencing data**

| Sample | Sequencing number | Sequencing data (Gb) | Reads count | Average reads length (bp) | Q30 (%) |
| --- | --- | --- | --- | --- | --- |
| H1 | m64032_191214 | 997 | 2894862 | 9376 | 99.99 |
| H2 | 21TW01445 | 896 | 2857696 | 10455 | 99.99 |
| H3 | 21TW01456 | 350 | 775983 | 13998 | 99.99 |
| H4 | 21TW01457 | 670 | 2121254 | 8948 | 99.99 |
| H5 | 21TW01460 | 202 | 489517 | 11662 | 99.99 |
| H6 | 21TW01658 | 825 | 2858457 | 7068 | 99.99 |
| H7 | 21TW01664 | 845 | 2283393 | 13371 | 99.99 |
| In total |  | 4785 | 14281162 |  |  |
