# Supplementary material for: PacBio sequencing of human fecal samples uncovers the DNA methylation landscape of 22 673 gut phages
Source: Nucleic Acids Res. 2023 Oct 30;51(22):12140–9. doi: 10.1093/nar/gkad977 (PMC10711547; doi:10.1093/nar/gkad977)
Supplement: gkad977_supplemental_files [file gkad977_supplemental_files.zip › Supplementary Figures.pdf]

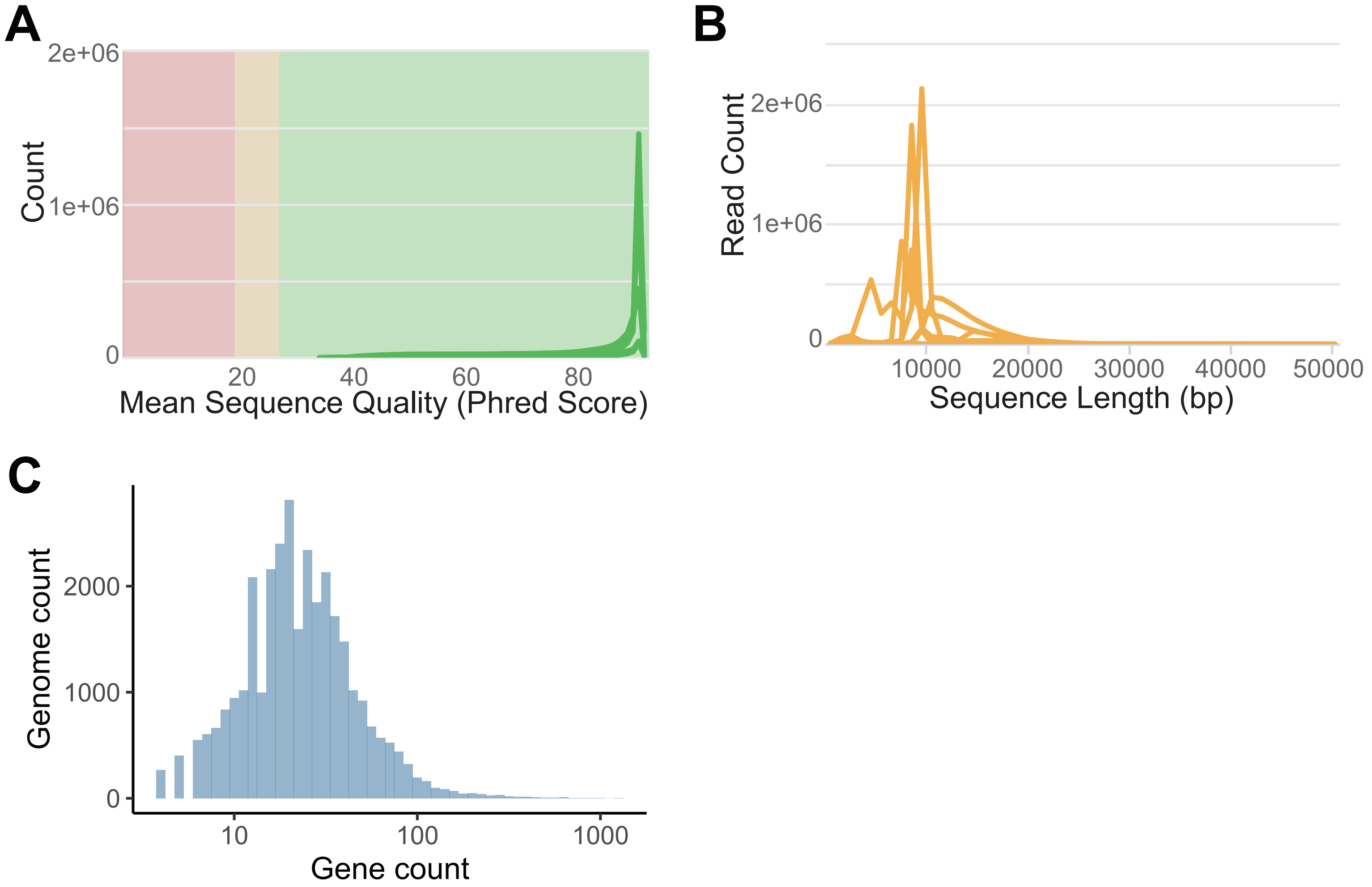

S Figure 1. PacBio sequencing data quality and virus genome assembly result. (A) the distribution of PacBio sequence quality score in each sample; **(B)** the distribution of PacBio sequence length in each sample; **(C)** the distribution of gene count in assembled genomes.

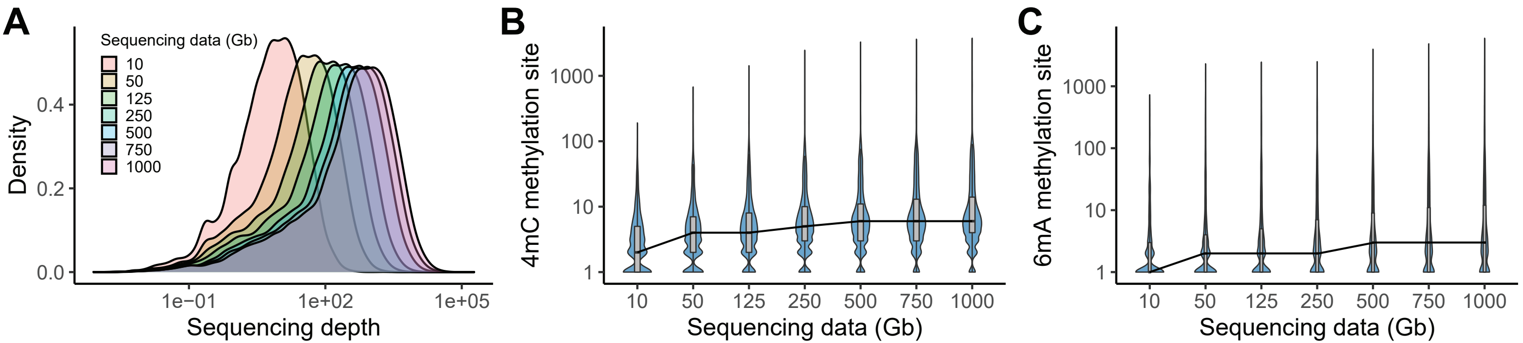

S Figure 2. Methylation detection under different amounts of sequencing data. (A) the distribution of sequencing depth under different amounts of sequencing data; **(B)** the number of 4mC and **(C)** 6mA methylation site detected under different amounts of sequencing data.

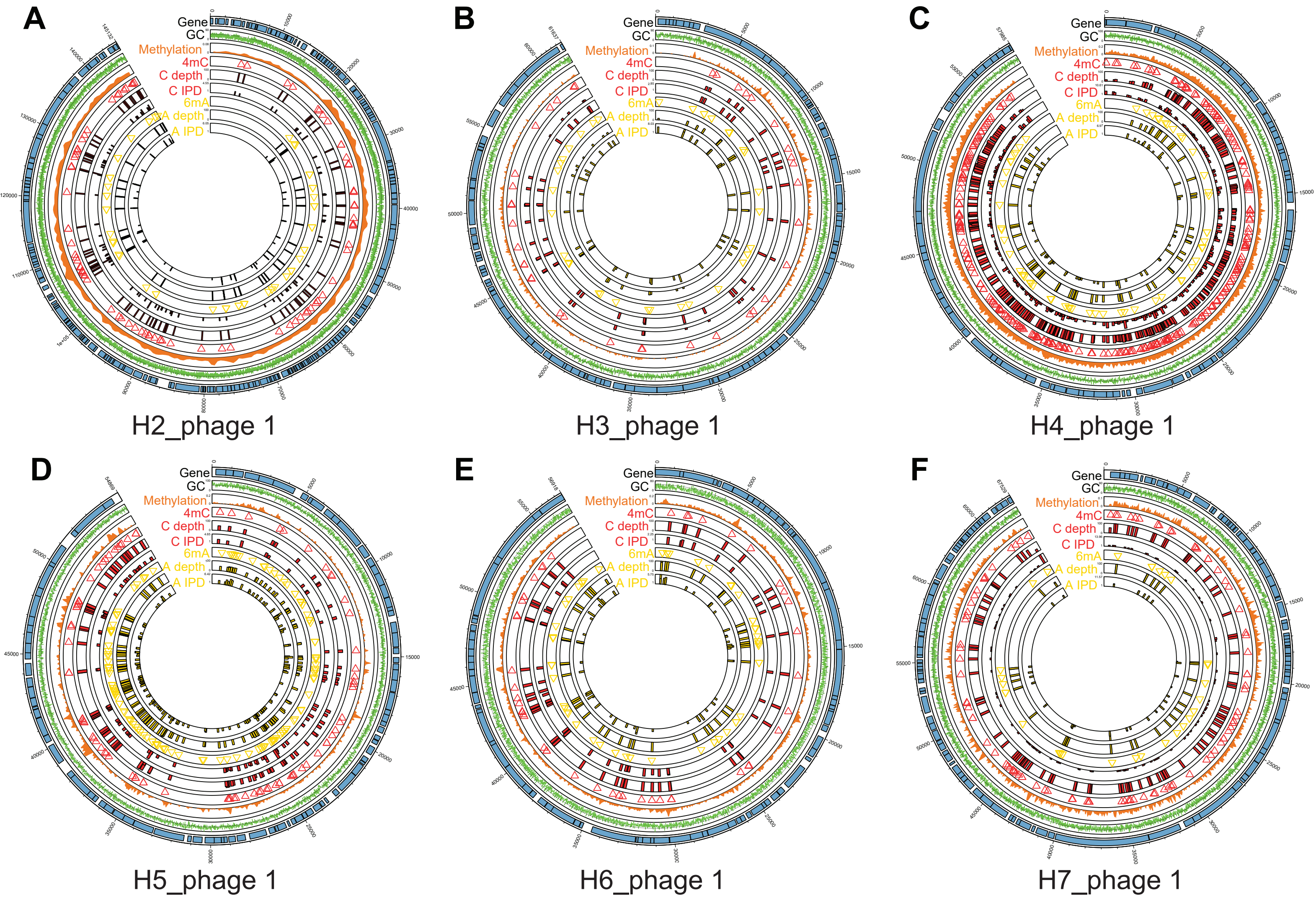

S Figure 3. Phage genome methylation maps. From inside to outside: 6mA inter-pulse duration (IPD), 6mA coverage depth, 6mA methylation site, 4mC IPD, 4mC coverage depth, 4mC methylation site, total methylation site, GC content and coding gene.

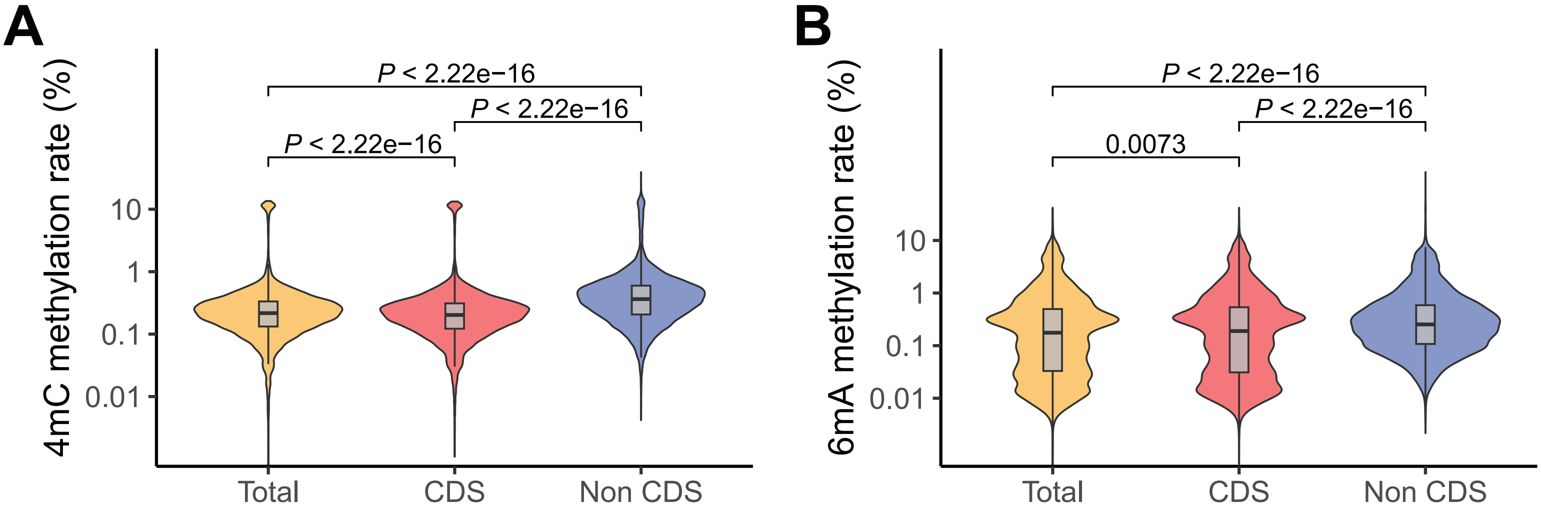

S Figure 4. The 4mC (A) and 6mA (B) methylation rates of CDS and non-CDS regions in phage genomes.

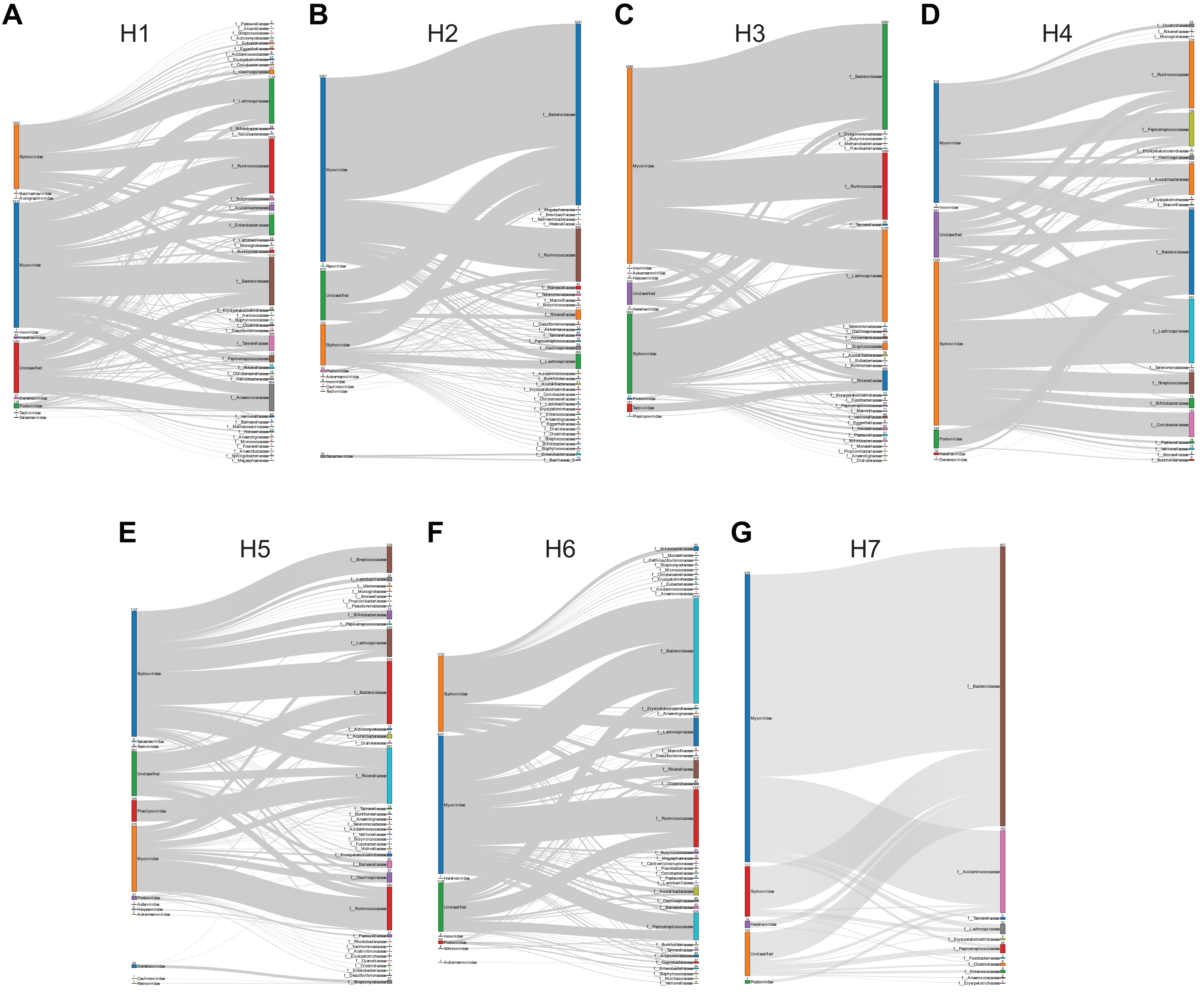

S Figure 5. The predicted hosts of phages in each sample. Phages (left) and predicted hosts (right) were connected by lines in each panel.

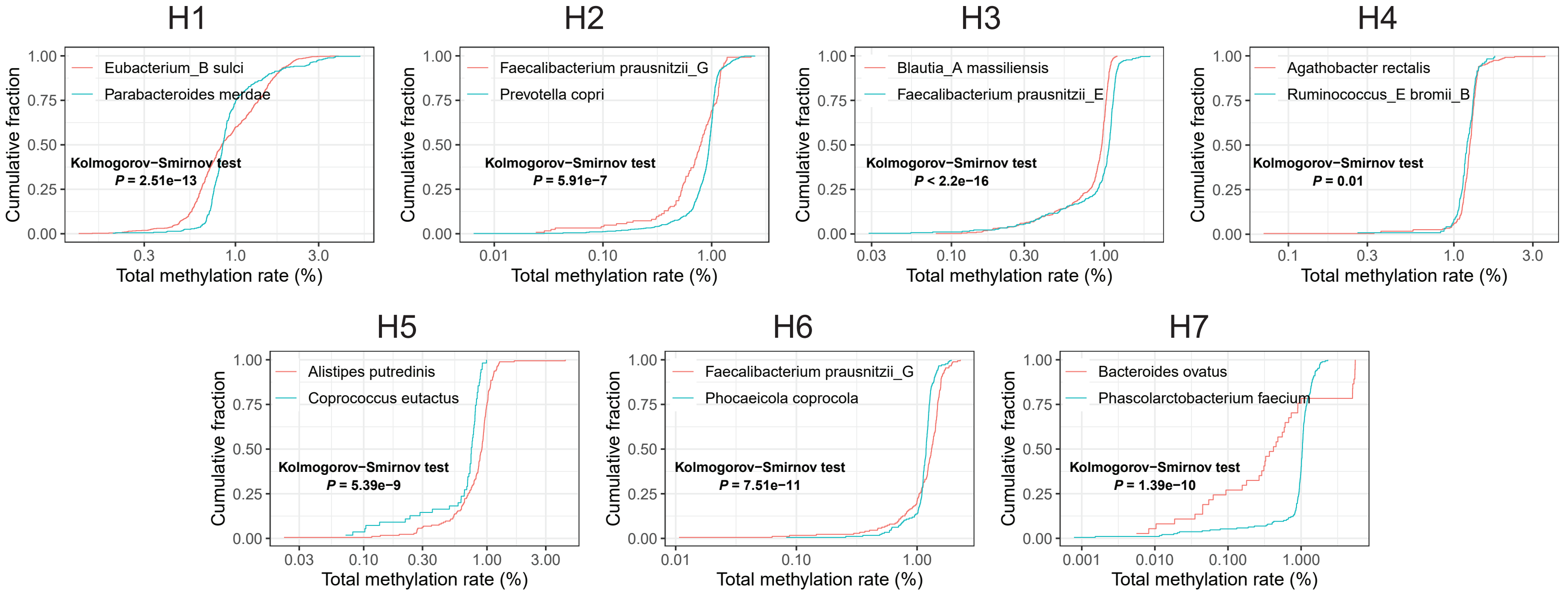

S Figure 6. Phages from different hosts had significantly different total methylation rate in each sample. The cumulative distribution analysis was performed to compare differences in the total methylation rates between phages from the top 2 hosts in each sample. The significant differences in total methylation rates were evaluated using the Kolmogorov-Smirnov test.

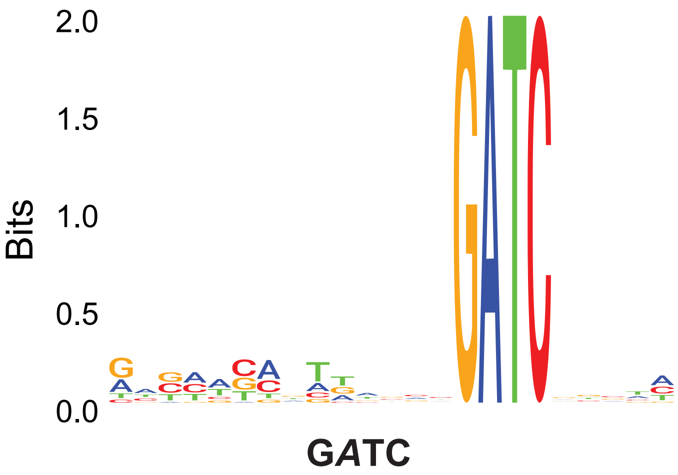

S Figure 7. The main 6mA methylation motif of rha antirepressor gene was GATC. Italic bold represents the methylated base.
